# Clinical and molecular spectrum of Niemann-Pick disease type C patients in India

**DOI:** 10.3389/fped.2026.1950899

**Published:** 2026-09-04

**Authors:** Jayesh Sheth, Aadhira Nair, Riddhi Bhavsar, Pankti Jasani, Niyati Saini, Aditi Baluni, Vykuntaraju K. Gowda, Mamta Muranjan, Ashish Bavdekar, Abhijeet Botre, Sandeep Kadam, Sheela Nampoothiri, Anupkumar P. Rawool, Chaitanya A. Datar, Nishant Dharsandia, Shruti Bajaj, Anupriya Kaur, Seema Kapoor, Dhaval Solanki, Siddharth M. Shah, Mahesh Kamate, I. C. Verma, Sushma K. Suresh, Vrajesh Udani, Rutuja Badge, Prachi Karnik, Soumya Mercy, Frenny Sheth, Harsh Sheth

**Affiliations:** 1 FRIGE’S Institute of Human Genetics, Ahmedabad, India; 2 Department of Pediatric Neurology, Indira Gandhi Institute of Child Health, Bangalore, India; 3 Department of Pediatrics, K.E.M Hospital, Mumbai, India; 4 Department of Pediatrics, K.E.M Hospital, Pune, India; 5 Department of Pediatric Genetics, Amrita Institute of Medical Sciences and Research Centre, Amrita Vishwa Vidyapeetham, Kochi, India; 6 Sahyadri Hospital, Hadapsar, Pune, India; 7 Bharati Hospital and Research Centre, Dhankawadi, Pune, India; 8 Hem Onc Care Center, Rajkot, India; 9 The Purple Gene Clinic, Simplex Khushaangan, Malad West, Mumbai, India; 10 Postgraduate Institute of Medical Education and Research (PGIMER), Chandigarh, India; 11 Division of Genetics and Metabolism, Department of Pediatrics, Maulana Azad Medical College, Delhi, India; 12 Nirmal Mantra Children’s Hospital, Bhavnagar, India; 13 Royal Institute of Child Neurosciences, Vastrapur, Ahmedabad, India; 14 KLES Prabhakar Kore Hospital, Belgaum, India; 15 Centre of Medical Genetics, Sir Ganga Ram Hospital, New Delhi, India; 16 St. John’s Medical College, Bangalore, India; 17 P. D. Hinduja National Hospital and Medical Research Center, Mumbai, India; 18 Department of Pediatrics, Lokmanya Tilak Municipal Medical College & General Hospital (Sion Hospital), Mumbai, India; 19 Sri Venkateswara Ramnarayan Ruia Government General Hospital, Tirupati, India

**Keywords:** Niemann-Pick disease type C, novel variant, *NPC1*, *NPC2*, smMIP

## Abstract

**Background:**

Niemann–Pick disease type C (NPC) is a rare autosomal recessive lysosomal disorder caused by pathogenic variants in *NPC1* or *NPC2*, resulting in impaired intracellular cholesterol trafficking and progressive neurovisceral disease. Although the clinical and molecular spectrum of NPC has been well characterized in several populations, data from India remain limited. The present study aimed to describe the clinical and molecular characteristics of NPC in Indian patients and to expand the mutational spectrum of the disease.

**Methods:**

We conducted a retrospective multicentre study of 28 patients with NPC diagnosed between 2010 and 2026 based on clinical, biochemical, and molecular investigations. Filipin staining was done in two patients. Plasma chitotriosidase activity (*n* = 21), *β*-glucosidase (*n* = 16) and sphingomyelinase activities (*n* = 21) were measured in plasma sample and leukocytes respectively using fluorometric enzyme assays. Molecular testing included clinical exome sequencing and Sanger sequencing in 2 patients and 1 patient, respectively while single-molecule molecular inversion probes (smMIP)-based targeted sequencing assay was performed in 25 patients. *In-silico* tools were used to predict the effect of novel variants on protein function and structure.

**Results:**

Hepatosplenomegaly was the most common presenting feature, followed by developmental delay, neuroregression, ataxia, seizures, and vertical supranuclear gaze palsy. Importantly, nearly one-third of patients presented with isolated visceral manifestations in the absence of neurological involvement. Molecular analysis identified 32 variants (27 in *NPC1* and 5 in *NPC2*), including 11 novel variants and two exon-level deletions. More than 60% of diagnoses were established during the final four years of the study following implementation of the FRIGE–Sanofi DISHA Program, highlighting the impact of improved access to affordable biochemical and molecular testing for lysosomal storage disorders. The indigenous smMIP assay enabled simultaneous detection of single nucleotide variants, small insertions/deletions, and exon-level copy number variants within a single cost-effective workflow. Structural analysis predicted that novel missense variants affect functionally critical domains of NPC1, resulting in disruption of protein stability, cholesterol trafficking, and lysosomal function.

**Conclusion:**

The present study provides a comprehensive characterization of the clinical spectrum and molecular heterogeneity of NPC in a large cohort of patients of Indian origin.

## Introduction

Niemann-Pick type C (NPC) is a rare, autosomal recessive, neurodegenerative lysosomal storage disorder resulting from faulty intracellular lipid trafficking. Based on earlier studies, NPC has a birth prevalence of approximately 1 per 120,000 live births (1, 2). Approximately 95% of affected individuals have mutations in *NPC1*, while *NPC2* mutations account for the remaining 5% (1–3). Impaired function of these proteins leads to lysosomal accumulation of unesterified cholesterol and secondary storage of sphingolipids, ultimately causing progressive neurovisceral disease.

The clinical manifestation of NPC is varied and is usually classified according to the age of neurological onset. Neonates commonly present with prolonged cholestatic jaundice and hepatosplenomegaly, whereas older children develop progressive neurological manifestations including developmental delay, neuroregression, cerebellar ataxia, vertical supranuclear gaze palsy, seizures, and cognitive decline (1, 2). Importantly, visceral manifestations often precede neurological involvement by several months or years, providing a critical window for diagnosis and genetic counselling (1, 2).

The mutational spectrum of *NPC1* is extensive, with over 486 disease-causing variants documented in the Human Gene Mutation Database (HGMD), while for *NPC2*, only 27 mutations have been reported (3). Recurrent mutations in *NPC1* includes the p.Ile1061Thr (c.3182T>C) variant, commonly found in NPC1 patients of Western Europe and the p.Pro1007Ala (c.3019C>G) variant, which is strongly associated with adult-onset disease (3–5). The p.Glu20Ter nonsense mutation in *NPC2* remains the most commonly reported pathogenic allele globally (3).

While multiple reports regarding the founder variants and other disease-causing variants in various populations are available (2–6), the genetics of *NPC1* and *NPC2* remains largely unknown in India. Comprehensive data describing genotype–phenotype correlations and the molecular architecture of Indian patients are lacking, despite the country's large and genetically diverse population. Expanding these data is important for improving clinical recognition, establishing population-specific variant databases, and facilitating accurate molecular diagnosis.

The aim of this study is to provide the first report pertaining to clinical and molecular characterization of NPC patients of Indian origin diagnosed over a 16-year period.

## Materials and methods

### Patient cohort details

The present study comprised 28 patients referred by clinicians from across India, including 13 patients from Maharashtra, 6 from Karnataka, 4 from Gujarat, 2 from New Delhi and one case each from Kerala, Chandigarh and Andhra Pradesh. These cases were pooled over a 16-year period (2010–2026). The majority cases were referred for the diagnosis of a Gaucher and Niemann Pick A/B disease and only 2 were referred specifically for the investigation of NPC. The report also includes 11 previously reported patients (7, 8). The study was approved by the Ethics committee at FRIGE's Institute of Human Genetics (Reg No-E/13237) in accordance with the Helsinki Declaration of 1975. Informed consent for investigation and publication of the data was obtained from the parents of all patients.

### Clinical and biochemical evaluation

**Figure 1:**
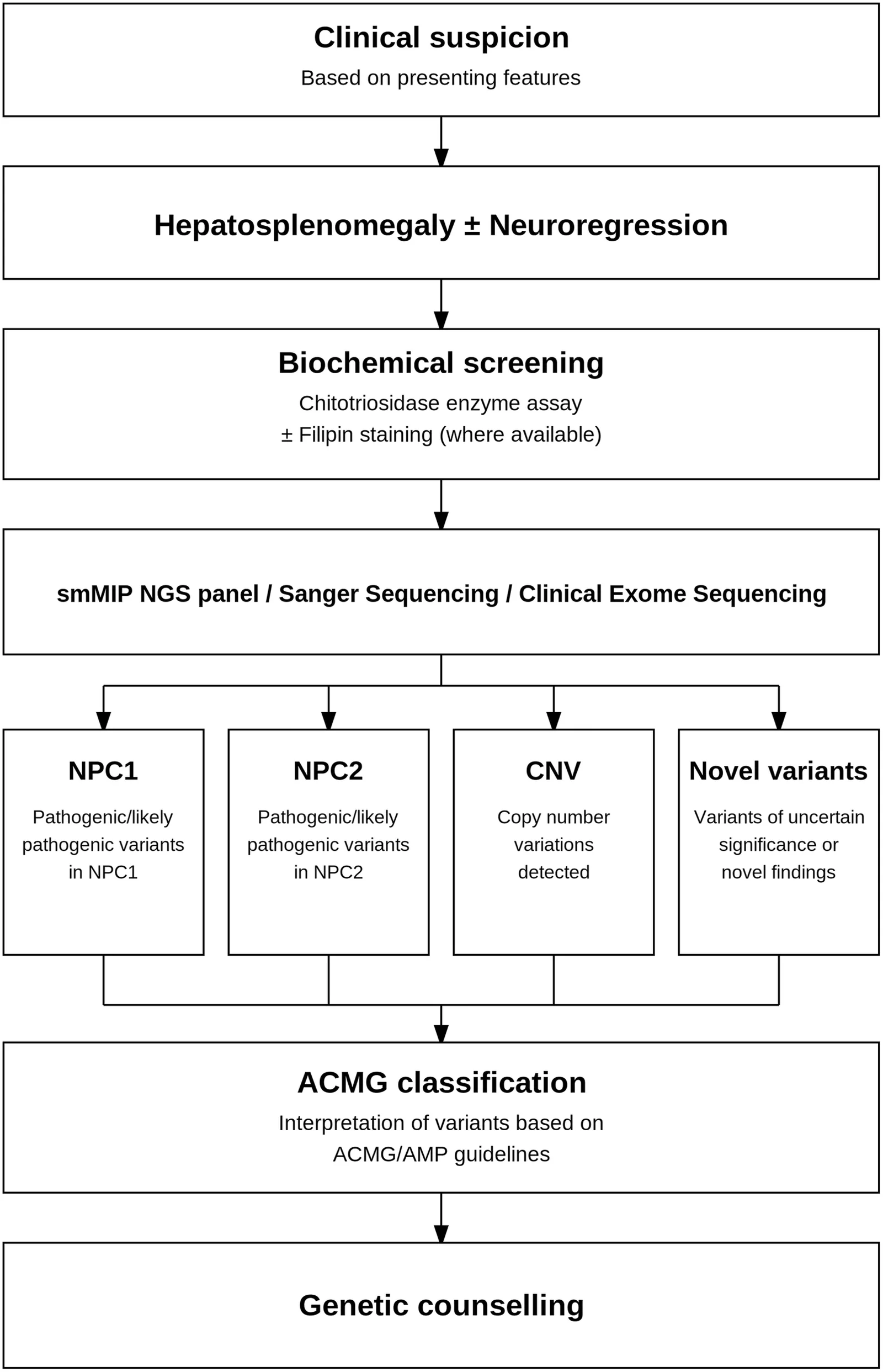
Overview of the diagnostic workflow used in this study.

Clinical information, demographic characteristics, family history, and laboratory findings were collected retrospectively from clinical records. Peripheral blood sample (3–4 mL) collected in an EDTA vial from all patients was subjected to plasma separation. For performing biochemical analysis, leukocytes were separated from whole blood as described previously (9) and stored at −20 °C for maximum 2 days. Figure 1 provides overview of the diagnostic workflow used in this study.

### Plasma chitotriosidase testing and filipin staining

Assessment of chitotriosidase enzyme activity in plasma sample was done using a 4-MU (4-methylumbelliferryl β-D-N,N,N′,N″-triacetylchitotrioside) substrate and assay conditions as described previously (10). Filipin staining was performed using cultured skin fibroblasts of two patients (P11 and P13) as described earlier (11, 12).

### Enzyme study

Fluorometric assays were performed to measure the activities of β-glucosidase and sphingomyelinase in leukocyte samples using 4-methylumbelliferyl-β-D-glucopyranoside and Hexadecarbonylamino-p-nitro phenyl phosphorylcholine substrate respectively (13, 14).

### DNA extraction and storage

Peripheral blood samples were collected in EDTA tubes, and genomic DNA was extracted using the salting-out method (Miller, Dykes and Polesky, 1988). The extracted DNA was quantified using the QIAxpert spectrophotometer (Qiagen, Hilden, Germany), and its quality was assessed by electrophoresis on a 2% agarose gel. The DNA samples were stored at −20 °C until further analysis.

### Molecular diagnosis

Molecular diagnosis was established using one of three complementary approaches according to sample availability and period of recruitment.

Of 28 patients, genomic DNA of 25 patients with a strong clinical suspicion of Gaucher disease, Niemann-Pick disease A/B or Niemann-Pick disease C were subjected to targeted sequencing using single-molecule molecular inversion probes (smMIP)-based NGS assay as described previously (7). Briefly, a targeted capture of genomic regions encompassing 23 genes associated with 29 common lysosomal storage disorders was performed using 100 ng genomic DNA as input. The resulting libraries were pooled in equimolar concentrations, purified using AMPure XP beads, and sequenced on the Illumina MiSeq platform (Illumina, USA) using custom sequencing primers with 2 × 156 bp paired-end reads. Sequencing was performed at a mean target coverage of ∼200x.

Clinical-exome sequencing (CES) was performed for two patients (P19 and P25) using custom capture kit that targets the exon and splice-site regions of 8,332 genes associated with known inherited diseases. Paired-end sequencing was carried out on Illumina HiSeq 2,500 platform (Illumina, USA) with a mean coverage of 100x. Molecular diagnosis for one patient (P24) was performed using Sanger sequencing of the *NPC2* gene (Supplementary File S1).

### NGS data analysis pipeline

FASTQ files generated from smMIP-NGS and CES were aligned to the GRCh37/hg19 reference genome using BWA-MEM v0.7.12 (15). Single nucleotide variants (SNVs) and indels were called using GATK HaplotypeCaller v4.1.2, following base quality recalibration and GATK best-practice workflows (16). DECoN v1.0.1 was used for detection of copy number variants (CNVs), including single-exon level CNVs in smMIP-NGS data. For detection of CNVs from CES data, ExomeDepth (v1.1.10) was used (16). CNVs identified from smMIP-NGS data using DECoN were independently validated by end-point PCR. The exon-level deletions identified in patients P2 and P27 were confirmed by amplification-based testing using primers targeting the respective deleted regions (Supplementary File S2).

Variant annotation and prioritization were performed using Exomiser v12 (17), incorporating HPO-based phenotype matching and data from multiple *in silico* prediction tools (SIFT, PolyPhen-2, MutationTaster, CADD, REVEL) and databases (dbSNP, gnomAD, ClinVar). Variants were annotated according to the Human Genome Variation Society (HGVS) nomenclature using reference transcripts NM_000271.5 (*NPC1*) and NM_006432.5 (*NPC2*) against the GRCh37/hg19 genomic build. Variants with MAF >1% in the 1,000 Genomes, TopMed, and gnomAD databases were excluded. Variants were prioritized based on their potential relevance to the patient's phenotype, including non-synonymous and splice-site variants with a read depth >20x. Intronic variants located close to exon-intron boundaries were also reviewed as potential splice-region variants, particularly when supported by their rarity, predicted functional consequences, disease-associated evidence, and phenotypic concordance. Candidate variants were interpreted according to ACMG-AMP guidelines and ClinGen framework (44, 46).

### *In silico* analysis

The effect of missense variants identified in the study were predicted using various pathogenicity prediction tools. These included MutationTaster (http://www.mutationtaster.org), PROVEAN (http://provean.jcvi.org), PolyPhen-2 (http://genetics.bwh.harvard.edu/pph2), FATHMM and Mutation Assessor (http://www.mutationassessor.org). Pathogenicity was also assessed using AlphaMissense, with scores >0.5 indicating likely pathogenic effects.

In order to explore the structural consequences of selected novel variants, protein modelling was performed using the experimentally resolved NPC1 crystal structure (PDB ID: 6W5S). Amino acid substitutions were introduced using PyMOL, while predicted effects on protein stability and residue interactions were evaluated using DynaMut2 (18) and the Arpeggio server (19). Protein stability changes upon mutation were quantified using ΔΔG (kcal/mol), where a negative value indicates a destabilising effect on the protein structure. Detailed methodology has been described in Supplementary File S1.

## Results

### Study cohort

The present study describes 28 patients diagnosed with Niemann-Pick type C, comprising 17 males (60.7%) and 11 females (39.3%). The age at diagnosis of the patients ranged from 1.5 months to 10 years. Consanguinity was documented in eight families (28.6%), while no consanguinity was reported in 15 families (53.6%). For five families, consanguinity details were not available. There was a family history suggestive of a similar disorder and sibling death in five patients.

### Clinical details

**Figure 2:**
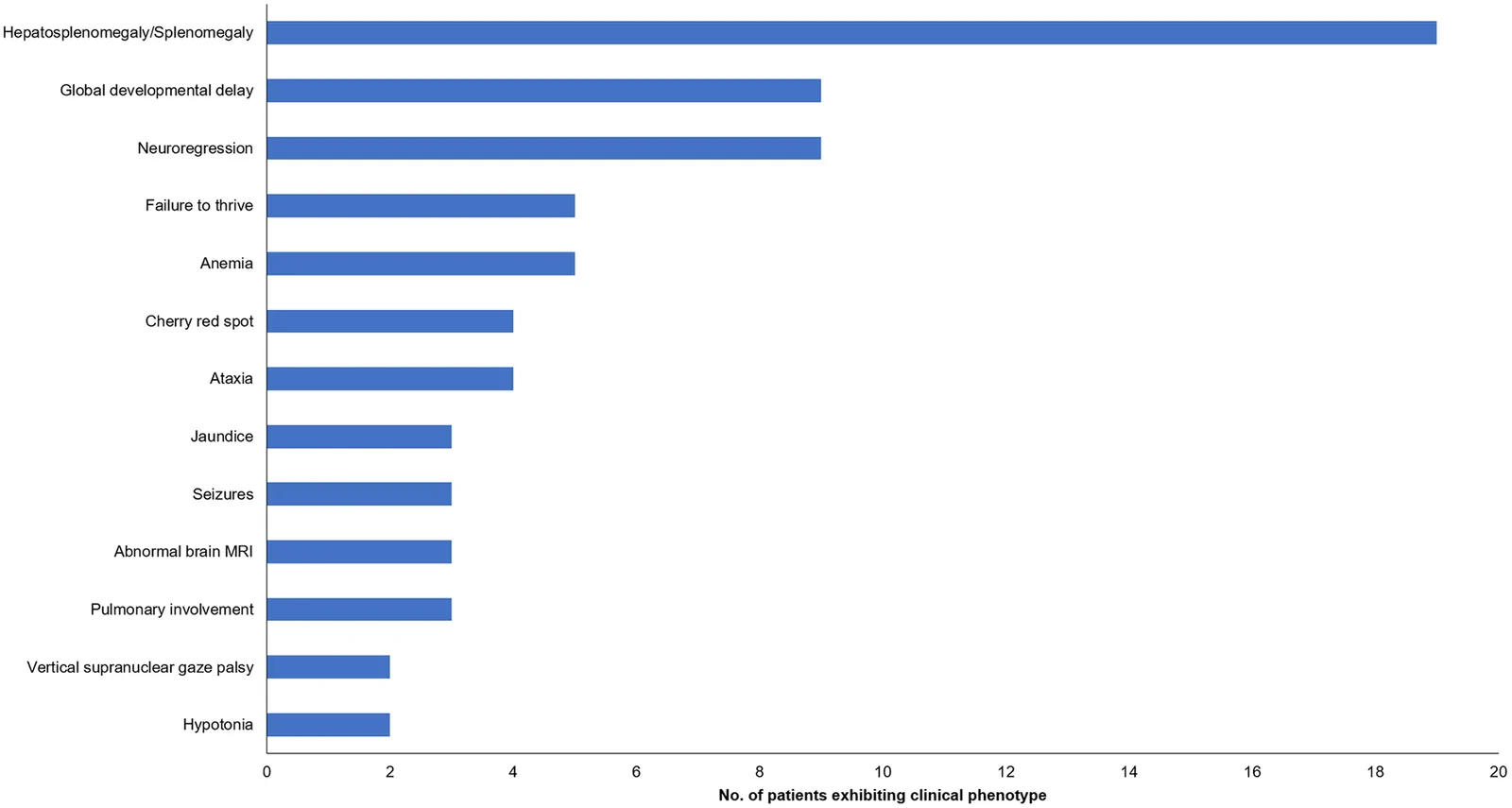
Frequency of clinical phenotype observed in the NPC patient cohort. The graph shows the percentage of patients having a specific symptom.

The most common clinical signs noted among patients at the time of presentation were hepatosplenomegaly/splenomegaly (*n* = 19/28), global developmental delay (*n* = 9/28), neuroregression (*n* = 7/28), failure to thrive (*n* = 5/28), anemia (*n* = 5/28), cherry red spot (*n* = 4/28), ataxia (*n* = 4/28), jaundice (*n* = 3/28) and seizures (*n* = 3/28). Pulmonary involvement was observed in three patients, while vertical supranuclear gaze palsy, and hypotonia were each noted in two patients. Abnormal brain MRI findings were reported in three patients and included delayed myelination, cerebral atrophy, and cerebellar atrophy. Bone marrow examination was performed in four patients. Foamy macrophages suggestive of Niemann–Pick disease were identified in two patients, whereas Gaucher cells were observed in the remaining two patients. At the time of diagnosis, nine patients (32.1%) presented without neurological manifestations and exhibited predominantly visceral disease (Figure 2). Tables 1, 2 provides clinical signs, biochemical findings, and genetic variant information for all 28 patients included in the study.

**Table 1:** Clinical, biochemical and molecular data of 21 patients diagnosed with NPC in the study.

Patient ID.	Age at diagnosis/gender	Consanguinity/family history	Region/state	Systemic manifestations	Neurological involvement	Plasma chitotriosidase (nmol/h/mL plasm)	*β*-glucosidase (nmol/h/mg protein)	Sphingomyelinase (nmol/h/mg protein)	Gene	Codon change (amino acid change)/location	Zygosity	Novel or reported	ACMG-AMP/ClinGen variant classification
P1	6 months/F	Third degree consanguinity/No	Maharashtra	Hepatosplenomegaly, bone marrow study-foamy Niemann-Pick cells	Hypotonia	10,847.3	not done	0.46	*NPC1*	c.352_353del (p.Gln119Valfs*8)/Exon 4	Homo	Reported	Pathogenic (PM3_Very strong; PVS1_Very strong; PM2_Moderate)
P2	2 Y 8 months/M	Third degree consanguinity/No	Karnataka	Splenomegaly	Global developmental delay with neuroregression, ataxia, seizures	55.2	5.8	2.7	*NPC1*	chr18:21148787_21148962del/Exon 4	Homo	Novel	Likely Pathogenic [1A (0.00); 2E (+0.90), 3A (0.00); overall score-0.91]
P5	3 Y/F	Third degree/death of one male sibling at 3 months,? jaundice	Gujarat	None	Fundus-cherry red spot, poor speech development, failure to thrive, sit with support, MRI-suggestive of delayed myelination	4,132.6	not done	0.9	*NPC1*	c.1326+1G>A/Intron 8	Homo	Novel	Pathogenic (PM3_Strong; PVS1_Very strong; PM2_Moderate)
P8	1 Y/M	Not available	Maharashtra	Splenomegaly, anemia	None	4,231.6	not done	1.2	*NPC1*	c.1819C>T (p.Arg607Ter)/Exon 12	Homo	Reported	Pathogenic (PM3_Very strong; PVS1_Very strong; PM2_Moderate)
P10	2 Y 6 months/M	No/one female sibling died at day 3, cause unknown	Karnataka	Moderate hepatosplenomegaly	Regression of milestones, coarse facial features-gum hypertrophy, growth retardation	not done	not done	not done	*NPC1*	c.2291C>A (p.Ala764Glu)/Exon 15	Homo	Novel	Pathogenic (PM3_Strong; PM1_Moderate; PP2_Supporting; PM2_Moderate; PM5_Moderate; PP3_Supporting)
P11	2.5 months/F	Not available	Delhi	Hepatosplenomegaly, jaundice	No details available	not done	not done	not done	*NPC1*	c.2533del (p.Val845Phefs*9)/Exon 17	Homo	Novel	Likely Pathogenic (PVS1_Very strong; PM2_Moderate)
P12	4 months/M	second degree consanguinity/no family history	Karnataka	Hepatosplenomegaly, pallor, jaundice, lung involvement, failure to thrive	Microcephaly, neurological manifestation	29.5	2.4	0.8	*NPC1*	c.2777C>T (p.Ala926Val)/Exon 18	Homo	Reported	Pathogenic (PM3_Very strong; PM1_Moderate; PP2_Supporting; PM2_Moderate; PM5_Moderate; PP3_Supporting)
P13	2 months/M	Not available	Kerala	Hepatosplenomegaly, jaundice	No details available	not done	not done	not done	*NPC1*	c.2767A>G (p.Ile923Val)/Exon 18	Homo	Reported	VUS (PM1_Moderate; PP2_Supporting; PM2_Moderate)
P16	7 Y/M	None	Maharashtra	Splenomegaly, liver failure	Cherry red spot, seizures, ataxia, oculomotor palsy, motor regression	413.2	1.2	2.1	*NPC1*	c.2873G>A (p.Arg958Gln)/Exon 19	Homo	Reported	Pathogenic (PM3_Very strong; PM1_Moderate; PP2_Supporting; PM2_Moderate; PM5_Moderate; PP3_Supporting)
P17	2 Y 9 months/F	Not available	New Delhi	Fatigue, pallor, enlarged spleen, bone marrow suggestive of Gaucher disease, Anaemia	Cherry red spot	1.2	4.7	not done	*NPC1*	c.2873G>T (p.Arg958Leu)/Exon 19	Homo	Reported	Likely Pathogenic (PM1_Moderate; PP2_Supporting; PM2_Moderate; PM5_Moderate; PP3_Supporting; PP5_Supporting)
P18	1 Y 6 months/M	None	Chandigarh	Hepatosplenomegaly	Global developmental delay	36.9	4.2	1.9	*NPC1*	c.2801G>A (p.Arg934Gln)/Exon 19	Homo	Reported	Pathogenic (PM3_Very strong; PM1_Moderate; PP2_Supporting; PM2_Moderate)
P19	3 Y/M	None	Gujarat	Hepatosplenomegaly	None	21,963.9	7.4	3.5	*NPC1*	c.3056A>G (p.Tyr1019Cys)/Exon 21	Homo	Reported	Pathogenic (PM3_Very strong; PM1_Moderate; PP2_Supporting; PM2_Moderate; PP3_Supporting)
P20	10 Y/M	None	Maharashtra	Hepatosplenomegaly, anaemia, thrombocytopenia	None	4,381.7	3.4	0.745	*NPC1*	c.3278C>T (p.Thr1093Ile)/Exon 22	Homo	Novel	Likely Pathogenic (PM1_Moderate; PP2_Supporting; PM2_Moderate; PP3_Supporting)
P21	1 month 15 days/F	Yes, one male sibling died at 3 months of age	Maharashtra	Hepatosplenomegaly, anemia	None	899.04	4.3	1.9	*NPC1*	c.3562del (p.Glu1188LysfsTer54)/Exon 23	Homo	Reported	Likely Pathogenic (PVS1_Strong; PM2_Moderate; PP5_Supporting)
P22	8 Y/M	Not available	Andhra Pradesh	Hepatosplenomegaly, anaemia, thrombocytopenia	No details available	5,239.6	4.3	0.8	*NPC1*	c.3508C>T (p.His1170Tyr)/Exon 23	Homo	Reported	Likely Pathogenic (PM1_Moderate; PP2_Supporting; PM2_Moderate; PP3_Supporting)
P23	5 months/M	Third degree consanguinity/No	Maharashtra	Abdominal distension since birth, café-au-lait spots on abdomen and forearm, hepatosplenomegaly	Hypotonia, cherry red spot, microcephaly, gait ataxia	478.1	7.9	2	*NPC1*	c.3754+3A>C/Intron 24	Homo	Reported	Likely Pathogenic (PM2_Moderate; PP3_Moderate; PP5_Strong)
P24	2 Y 9 months/M	Yes, no history	Maharashtra	Chronic grade II and III Dyspnoeic with hepatosplenomegaly, Bronchoalveolar lavage (BAL) showing a moderate number of foamy macrophages, bone marrow revealing foamy cells	None	not done	not done	1.1	*NPC2*	c.82+2T>C/Intron 1	Homo	Reported	Pathogenic (PM3_Strong; PVS1_Very strong; PM2_Moderate)
P25	1 Y 6 months/F	None	Maharashtra	Normal till 9 months of age, thereafter diagnosed with pneumonia and respiratory distress, interstitial pneumonitis, hepatosplenomegaly, failure to thrive, Lung biopsy: suggestive of alveolar proteinosis Liver biopsy: suggestive of NPC disease failure to thrive	Gross motor delay corresponding to 5–6 months	Elevated (done in another lab)	not done	Low (done in another lab)	*NPC2*	c.141C>A (p.Cys47Ter)/Exon 2	Homo	Reported	Pathogenic (PM3_Very strong; PVS1_Very strong; PM2_Moderate)
P26	4 Y/M	None	Gujarat	Inguinal hernia at 3 months, normal up to 3.5 years, thereafter splenomegaly, recurrent infections	Regression of milestone, neuroregression	12,167.2	1.3	1.9	*NPC2*	c.116_117dup (p.Ser40Ter)/Exon 2	Homo	Novel	Pathogenic (PM3_Strong; PVS1_Very strong; PM2_Moderate)
P27	3 Y/M	None	Maharashtra	None	Delayed development, psychomotor retardation, regressed cognitive and motor milestone, Brisk deep tendon reflexes (DTRs)	not done	not done	Low (done in another lab)	*NPC2*	Exons 2–3 deletion	Homo	Reported	Likely Pathogenic [1A (0.00); 2E (+0.90), 3A (0.00); overall score-0.91]
P28	8 months/M	No/one male sibling died suspected with LSD	Karnataka	Fatigue, pallor, enlarged spleen, bone marrow suggestive of Gaucher disease, Anaemia	Delayed milestone	2,765.1	0.9	2.3	*NPC2*	c.210_213dup (p.Ala72Glnfs*19)/Exon 3	Homo	Reported	Pathogenic (PM3_Strong; PVS1_Very strong; PM2_Moderate)

M, male; F, female; UK, Unknown; NCM, non-consanguineous marriage; Homo, homozygous.

Reference range for β-glucosidase: 4.0–32.0 nmol/h/mg protein, sphingomyelinase: 1.8–9.6 nmol/h/mg protein, plasma chitotriosidase: 1.3–80.0 nmol/h/mL plasma.

**Table 2:** Clinical, biochemical and molecular data of 7 patients with two heterozygous variants identified in *NPC1* gene.

Patient ID.	Age at diagnosis/Gender	Consanguinity/family history	Region/State	Systemic manifestations	Neurological involvement	Plasma chitotriosidase (nmol/h/mL plasm)	β-glucosidase (nmol/h/mg protein)	Sphingomyelinase (nmol/h/mg protein)	Gene	Codon change (amino acid change)/Location	Zygosity	Novel or reported	ACMG-AMP variant classification
P3	9 Y 4 months/M	NCM, no family history	Karnataka	None	Mild global developmental delay, neuroregression, quadriparesis, cerebellar ataxia, epilepsy, MRI-cerebral and cerebellar atrophy with white matter loss, EEG-multifocal epilepsy with quasiperiodic spike waves, fundus-tessellated background	not done	not done	not done	*NPC1*	c.955+3A>G/Intron 7	Likely Comp. Het	Novel	VUS (PM2_Moderate; PP3_Supporting)
										c.3182T>C (p.Ile1061Thr)/Exon 21		Reported	Pathogenic (PM3_Very strong; PS3_Supporting; PM1_Moderate; PP2_Supporting; PM2_Moderate)
P4	1 Y 6 months/M	NCM, no family history	Maharashtra	Hepatosplenomegaly and anaemia	None	32.6	1.3	2	*NPC1*	c.1132G>T (p.Val378Phe)/Exon 8	Likely Comp. Het	Novel	Pathogenic (PM3_Strong; PM2_Moderate; PM5_Moderate; PM1_Supporting; PP2_Supporting)
										c.2373+7T>C/Intron 5		Novel	VUS (PM2_Moderate)
P6	5 months/F	NCM, no family history	Maharashtra	Splenomegaly	Developmental delay, microcephaly, dystonia	354.6	6.3	1.4	*NPC1*	c.1408G>C (p.Ala470Pro)/Exon 9	Likely Comp. Het	Novel	VUS (PM2_Moderate; PP3_Supporting; PP2_Supporting)
										c.2509A>G (p.Ile837Val)/Exon 16		Reported	VUS (PM2_Moderate, PP2_Supporting)
P7	8 Y/F	NCM, one male sibling died at 20 Y of age, cause unknown	Maharashtra	None	Neurological manifestations (onset at 6y)	12,921.3	1.5	1.7	*NPC1*	c.1408G>C (p.Ala470Pro)/Exon 9	Likely Comp. Het	Novel	VUS (PM2_Moderate; PP3_Supporting; PP2_Supporting)
										c.2509A>G (p.Ile837Val)/Exon 16		Reported	VUS (PM2_Moderate, PP2_Supporting)
P9	9 Y/F	None	Gujarat	Hepatosplenomegaly, short stature	failure to thrive, delayed motor development, vertical gaze palsy	4,332.1	2.5	0.8	*NPC1*	c.2000C>T (p.Ser667Leu)/Exon 13	Likely Comp. Het	Reported	Pathogenic (PM3_Very strong; PM1_Moderate; PP2_Supporting; PM2_Moderate; PP3_Supporting)
										c.2974G>T (p.Gly992Trp)/Exon 20		Reported	Pathogenic (PM3_Very strong; PS1_Strong; PM5_Moderate; PM1_Moderate; PP2_Supporting; PM2_Moderate; PP3_Supporting)
P14	7 Y/F	None	Karnataka	None	Hypertonia, myoclonic jerks, vertical gaze palsy, cataplexy, MRI-mild cerebral atrophy, regression of milestone	not done	not done	not done	*NPC1*	c.2776G>A (p.Ala926Thr)/Exon 18	Likely Comp. Het	Reported	Pathogenic (PM3_Very strong; PM1_Moderate; PP2_Supporting; PM2_Moderate; PM5_Moderate; PP3_Supporting)
										c.2873G>A (p.Arg958Gln)/Exon 19		Reported	Pathogenic (PM3_Very strong; PM1_Moderate; PP2_Moderate; PM2_Moderate; PM5_Moderate; PP3_Supporting)
P15	2 Y/F	Third degree consanguinity/No	Maharashtra	Splenomegaly, feeding difficulty, progressive abdominal distention, mongoloid spots over the buttocks and back, failure to thrive, tall anterior forehead	Preterm delivery, motor delay of milestone, neuroregression after 18 months, oromotor dyskinesia, hyperkinesia, involuntary movements	8,755.3	4.4	0.7	*NPC1*	c.2673dup (p.Val892CysfsTer26)/Exon 18	Likely Comp. Het	Novel	Pathogenic (PM3_Strong; PVS1_Very strong; PM2_Moderate)
										c.3734_3735del (p.Pro1245ArgfsTer12)/Exon 24		Reported	Pathogenic (PM3_Very strong; PM2_Moderate; PVS1_Moderate)

M, male; F, female; UK, Unknown; NCM, non-consanguineous marriage; Comp het, compound heterozygous.

Reference range for β-glucosidase: 4.0–32.0 nmol/h/mg protein, sphingomyelinase: 1.8–9.6 nmol/h/mg protein, plasma chitotriosidase: 1.3–80.0 nmol/h/mL plasma.

### Biochemical investigations

Plasma chitotriosidase activity was measured in 21 patients, of whom 11 (52.4%) showed significantly elevated enzyme levels (Tables 1, 2). Filipin staining performed in cultured skin fibroblasts from two patients demonstrated intracellular accumulation of unesterified cholesterol, confirming the diagnosis of NPC. Leukocyte β-Glucosidase and sphingomyelinase activities were assessed in 16 patients with a clinical suspicion of Gaucher disease or Niemann-Pick disease. Of these, five patients showed reduced β-glucosidase activity, while five patients showed reduced sphingomyelinase activity. In an additional five patients with a strong clinical suspicion of Niemann-Pick disease, only sphingomyelinase activity was assessed, and was reduced in all cases.

### Molecular genetic analysis

Genetic study identified causative variants in the *NPC1* in 23 patients (82.1%) and in the *NPC2* gene in five patients (17.9%). Homozygous variants were identified in 21 patients (Table 1). In seven patients, two heterozygous variants were identified in the *NPC1* gene (Table 2). However, as the patients were lost to follow-up and parental DNA samples were not available, segregation study to delineate the inheritance mode and confirm compound heterozygosity could not be performed. For P14, based on the co-occurrence pattern in gnomAD, variants c.2776G>A and c.2873G>A are likely to be found on different haplotypes in most individuals in gnomAD, thereby suggesting compound heterozygosity.

**Figure 3:**
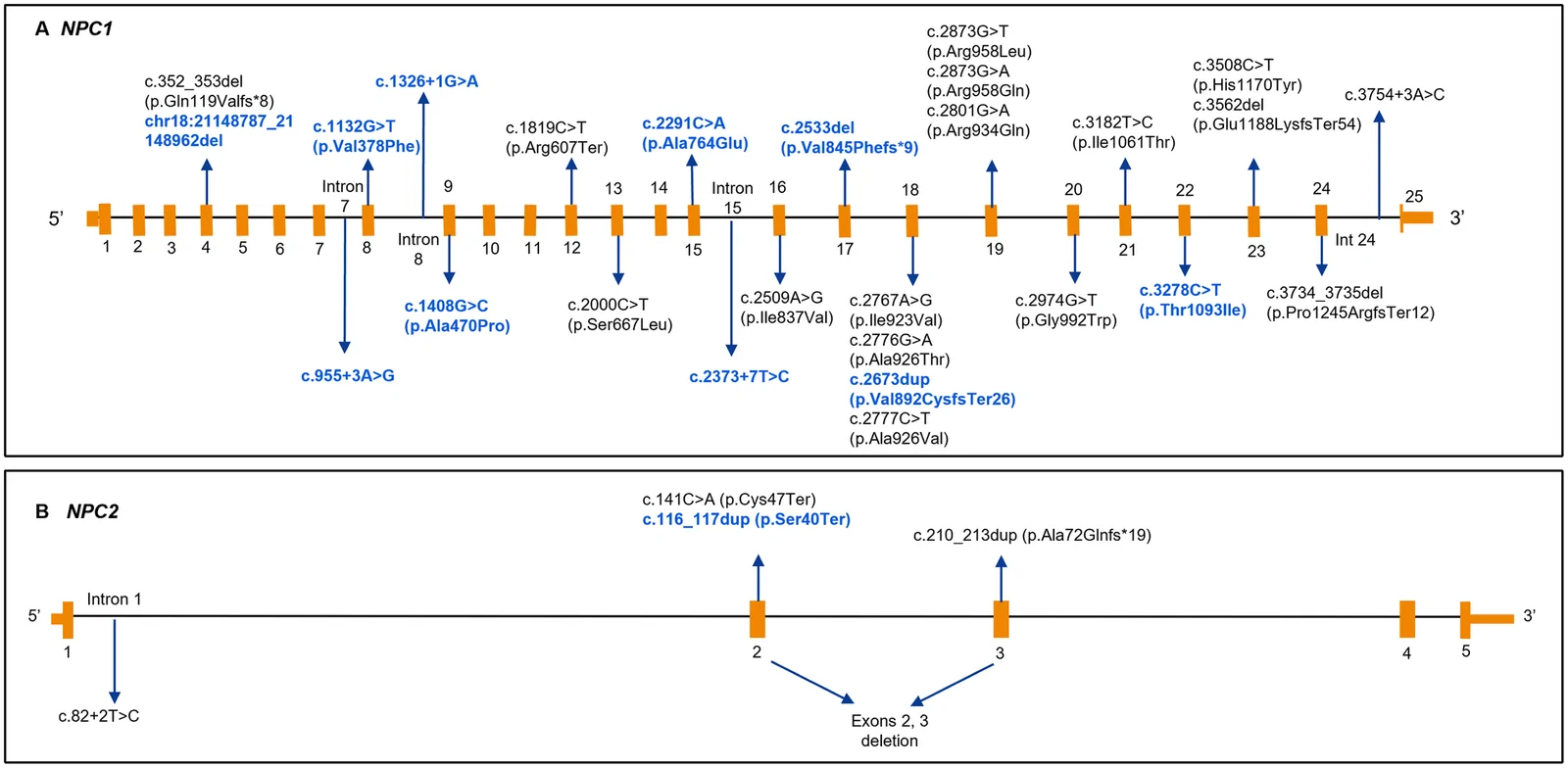
Schematic diagram of exons and introns of **(A)** NPC1 gene and **(B)** NPC2 gene. The variants harbored by the NPC patients included in the cohort are indicated. The novel variants reported in this study are marked in blue.

Overall, 32 variants (27 variants in *NPC1* and 5 variants in *NPC2*) were identified in 28 patients (Figures 3A,3B). Majority variants identified were missense (50%, *n* = 16/32), followed by frameshift (18.75%, *n* = 6/32) and splice-site variants (15.63%, *n* = 5/32). Nonsense variants were identified in three patients. Importantly, using the smMIP-NGS assay, we detected exon-level copy number variants in two patients: a homozygous exon 4 deletion in the *NPC1* gene in one patient and a homozygous exon 2–3 deletion in the *NPC2* gene in another. Both exon-level deletions were independently validated by end-point PCR, confirming the CNV calls obtained from smMIP-NGS assay (Supplementary File S2). We found 10 novel variants detected in *NPC1* while one novel variant was identified in *NPC2*. Other than the variants c.1408G>C and c.2509A>G in *NPC1*, detected in two unrelated patients, no other common variants were identified in the *NPC1* gene. Majority of the variants were located in exon 18 (*n* = 4/27) and exon 19 (*n* = 4/27) of the *NPC1* gene, whereas no common variant was identified in *NPC2*.

### *In-silico* analysis of novel variants

All novel missense variants were predicted to be diseases causing by multiple mutation prediction software including MutationTaster, PROVEAN, PolyPhen2, FATHMM, and CADD (Supplementary File S3). None of the novel variants were reported in gnomAD population database except c.1408G>C in *NPC1*, which was observed at an extremely low allele frequency in gnomAD of 0.0007%. Splicing effects were assessed for the canonical splice-site variant c.1326+1G>A and two splice-region variants (c.955+3A>G, c.2373+7T>C) using SpliceAI. The canonical donor-site variant c.1326+1G>A showed strong predicted splice disruption, with a SpliceAI donor-loss *Δ* score of 0.99. In contrast, the splice-region variants c.955+3A>G and c.2373+7T>C showed low SpliceAI scores (maximum *Δ* scores of 0.08 and 0.01, respectively) indicating no strong computational evidence for splice alteration. Furthermore, parental segregation and RNA-based functional studies could not be performed in the present cases as the patients were lost to follow-up. Hence, the phase of the variants and their effects on splicing could not be established. These two variants were therefore retained as candidate disease-associated splice-region variants.

### Protein modeling for structural-functional correlation of NPC1 variants

**Figure 4:**
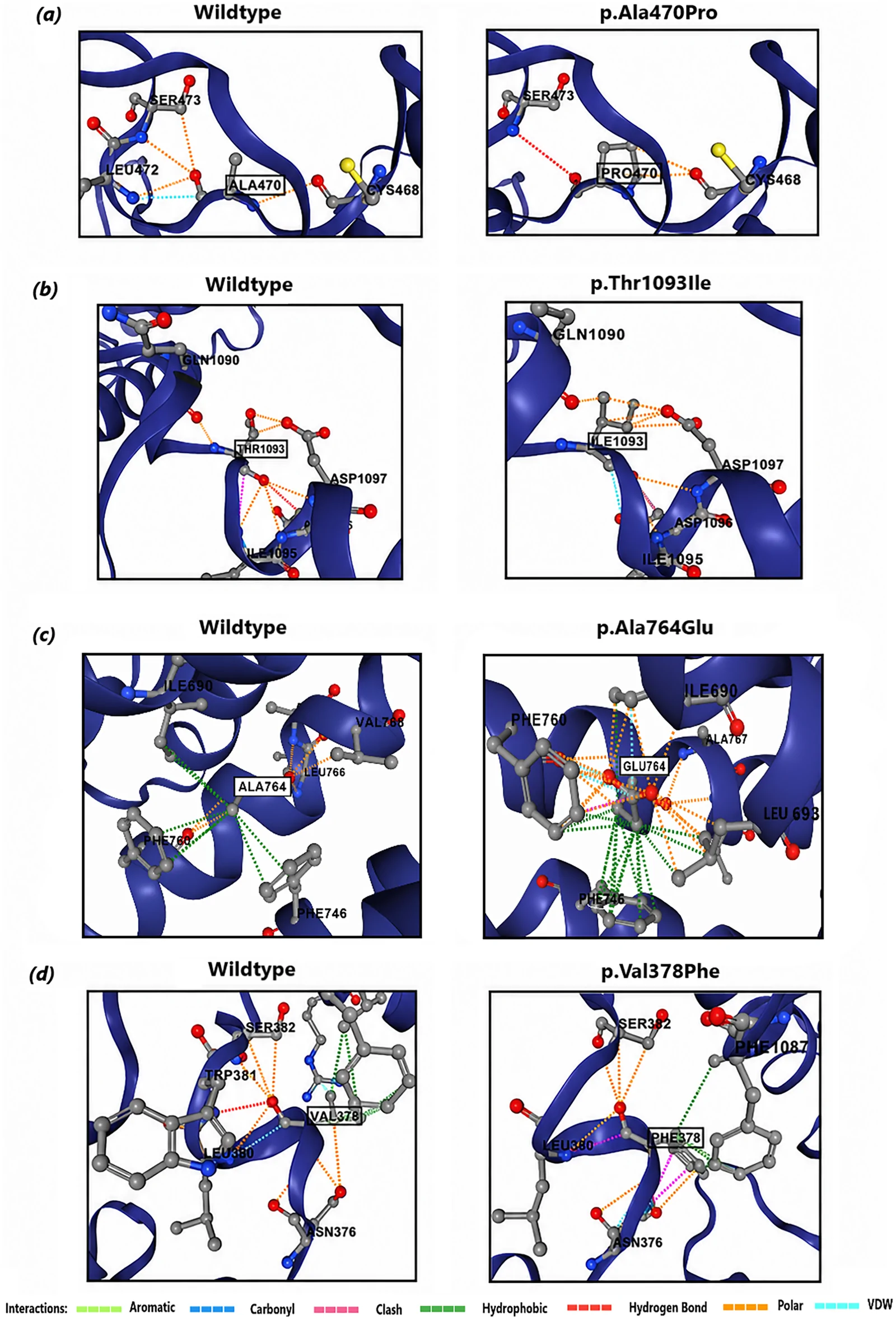
Wild-type and mutant NPC1 structures showing altered local interaction networks for **(a)** p.Ala470Pro, **(b)** p.Thr1093Ile, **(c)** p.Ala764Glu, and **(d)** p.Val378Phe. Left panels represent the wild-type NPC1 local residue interaction network; right panels represent the corresponding interaction network following mutation, as predicted by Dynamut2. Interaction types are indicated as hydrogen bonds, hydrophobic interactions, polar contacts, aromatic contacts, van der Waals interactions, carbonyl interactions, and steric clashes.

**Figure 5:**
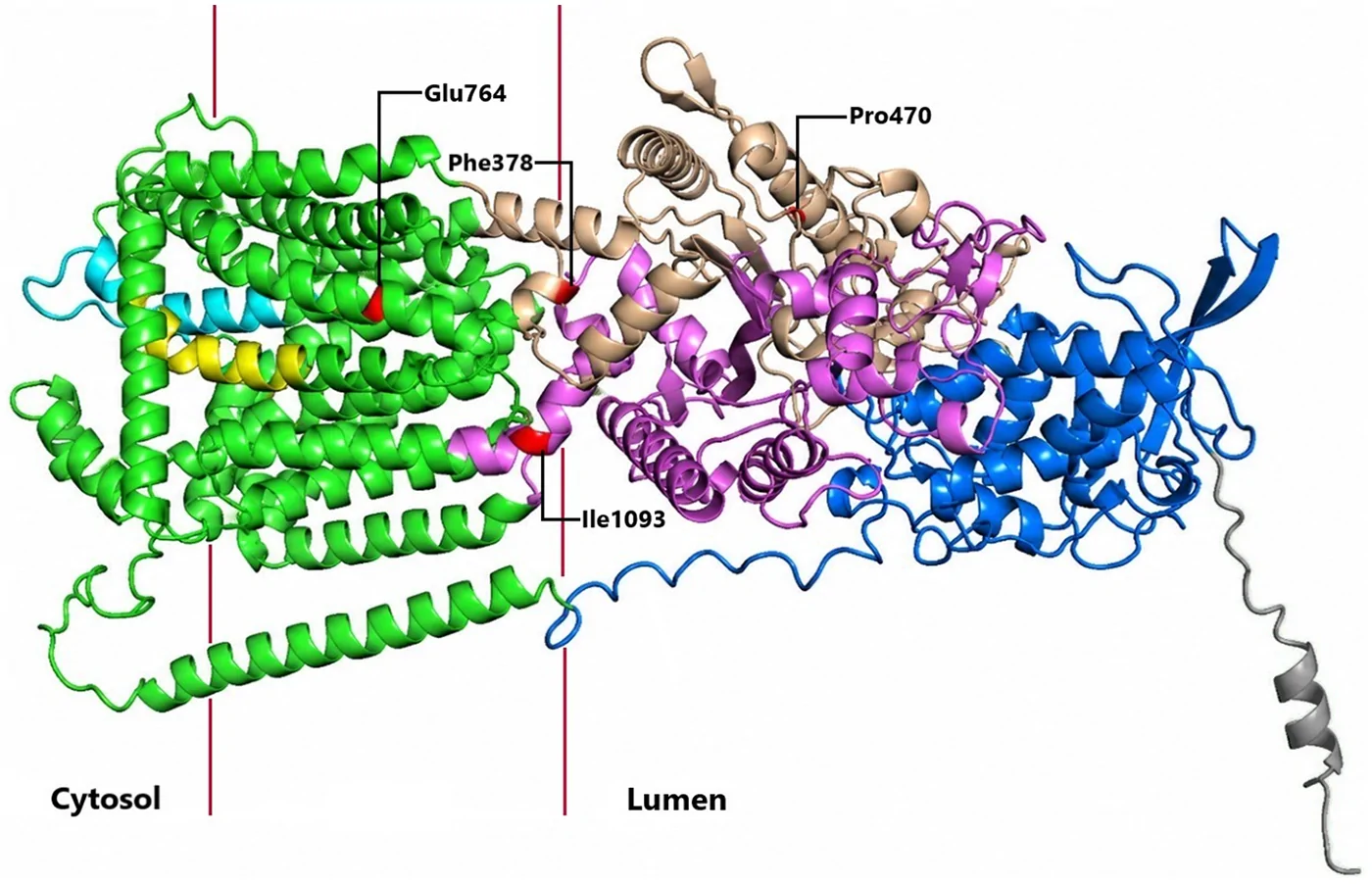
The structure of NPC1 (PDB: 6W5S) shows the location of the novel missense variants identified in this study, highlighted in red. Functional domains containing *NPC1* variants are indicated in different colors. MLD, middle luminal domain = wheat; CTD, C-terminal domain = violet; TM5 = cyan; Cytosolic loop = yellow; NTD, N-terminal domain = marine, while the remainder of the NPC1 structure, not corresponding to these specific domains of interest, is shown in green.

The NPC1 novel variants found in this study, p.Ala470Pro (c.1408G>C), p.Thr1093Ile (c.3278C>T), p.Ala764Glu (c.2291C>A) and p.Val378Phe (c.1132G>T), were tested for their effects on protein structure and function through *in-silico* analyses. Structural stability prediction using Dynamut2 showed a destabilizing effect on the NPC1 protein for all four variants, with ΔΔG values of −0.09 kcal/mol, −0.27 kcal/mol, −0.83 kcal/mol and −1.18 kcal/mol for p.Ala470Pro, p.Thr1093Ile, p.Ala764Glu and p.Val378Phe, respectively. Pathogenicity was assessed using AlphaMissense, which classified p.Ala470Pro, p.Thr1093Ile, and p.Ala764Glu as likely pathogenic, with scores of 0.672, 0.736, and 0.991, respectively, while p.Val378Phe gave an intermediate score of 0.499. MutPred2 further supported the pathogenic potential of p.Ala764Glu (score = 0.895) and p.Ala470Pro (score = 0.799), with p.Ala764Glu predicted to disrupt functional PROSITE and ELM motifs, and p.Ala470Pro associated with altered ordered interface and transmembrane protein properties, along with gain of a catalytic site and loss of disulfide linkage at Cys468. AlphaFold2-based MSA coverage analysis showed that p.Ala764Glu and p.Val378Phe are located in highly conserved regions of NPC1 supported by strong alignment depth, whereas p.Ala470Pro and p.Thr1093Ile lie in regions of comparatively reduced conservation, suggesting weaker evolutionary support for a deleterious effect at these two sites. Arpeggio analysis showed that the overall interaction network remained largely preserved for all four variants. Minor increases in the van der Waals clashes, weak polar contacts, and proximal interactions were observed indicating localized disruption rather than global structural perturbation. Representative structural models are shown in Figures 4, 5.

## Discussion

Niemann-Pick disease type C (NPC) is a rare, inherited cholesterol transport disorder caused by mutations in the *NPC1* or *NPC2* genes, resulting in impaired intracellular cholesterol trafficking (1, 2). Owing to its rarity, only a limited number of NPC patients have been reported across different populations, making it challenging to characterize the natural history of the disease and recruit patients for clinical studies. To address this, the International Niemann-Pick Disease Registry was established. They collected clinical data from 203 NPC patients across six European countries to improve understanding of the disease's natural history (20). In addition, several case reports and case series from different populations have expanded the knowledge of the clinical, biochemical, and molecular spectrum of NPC (4, 6, 21–23). However, reports describing Indian patients remain scarce (12, 24). To the best of our knowledge, the present study is the largest case series of NPC patients from India reported to date.

An important observation from our cohort was the marked increase in the number of NPC diagnoses during the last four years. Of the 28 patients included in this study, 17 (60.7%) were diagnosed between 2022 and 2026, whereas only 11 patients were diagnosed over the preceding 12-year period (2010–2021). This increase coincided with the implementation of the FRIGE-Sanofi DISHA Program in 2022, which provides free biochemical and molecular diagnostic testing for patients clinically suspected of five lysosomal storage disorders, including Gaucher disease, Niemann-Pick disease types A/B, Mucopolysaccharidosis I, Fabry disease, and Pompe disease. Thus, the availability of affordable targeted next-generation sequencing, coupled with increased clinical awareness and improved referral pathways, likely contributed to enhanced recognition of NPC. These findings highlight the importance of improving access to diagnostic programs in order to achieve molecular diagnosis of rare disorders such as NPC. Geographical distribution of our cohort revealed that the majority of patients originated from the western part of the country, particularly Maharashtra and Gujarat. This regional clustering is likely due to referral bias as most cases from these regions were received through the FRIGE-Sanofi DISHA program. Hence, this distribution does not reflect the true regional prevalence of NPC in India. Further studies are necessary to understand the epidemiology of NPC across the country.

The clinical presentation of NPC is highly heterogeneous, with patients developing a diverse range of systemic and neurological manifestations at different ages and with variable rates of disease progression (2, 25). This heterogeneity was also observed in our cohort (Table 1). Age at diagnosis for majority patients was between 2 months and <2 years (42.8%, *n* = 12/28), followed by 28.6% patients (*n* = 8/28) diagnosed between 2 and < 6 years. Notably, our cohort also included a substantial proportion of juvenile-onset cases (25%, *n* = 7/28) diagnosed between 6 and <15 years of age. No patients were diagnosed during adulthood. Although direct comparisons are limited because our data is based on age at diagnosis rather than age at disease onset, previous studies have reported a higher proportion of adult-onset NPC. For instance, Dardis et al. (3) reported a relatively high proportion of adult-onset NPC in their cohort, while data from the International NPC Registry showed that 20.8% of enrolled participants were adults (20). The absence of adult diagnoses in our cohort may reflect the under-recognition of adult-onset NPC in India. This is likely due to limited clinical awareness and the overlap of its neurological and psychiatric manifestations with more common adult-onset neurodegenerative disorders, such as Alzheimer's disease (26).

Consistent with previous reports, hepatosplenomegaly or isolated splenomegaly was the most frequent presenting feature (67.8% of patients), highlighting the importance of considering NPC in the differential diagnosis of children with unexplained visceral enlargement (20). Other systemic manifestations included failure to thrive (17.8%), jaundice (10.7%), and pulmonary involvement (10.7%). A study of NPC patients from Greece reported a comparable frequency of failure to thrive (20%) in their cohort (27). Prolonged neonatal jaundice accompanied by mild hepatosplenomegaly is one of the most common clinical presentations of NPC during the neonatal period. In most affected infants, jaundice resolves spontaneously by 3–4 months of age, whereas hepatosplenomegaly may persist to varying degrees (28). Because the present study was retrospective and longitudinal follow-up data were unavailable, detailed information on perinatal and neonatal manifestations could not be collected. As a result, neonatal jaundice may have been underreported, which could explain the relatively low frequency of jaundice observed in our cohort.

Developmental delay, neuroregression, ataxia, seizures, and vertical supranuclear gaze palsy constituted the predominant neurological manifestations. This is consistent with clinical observations documented in other NPC patient cohorts (20). Developmental milestone delay is one of the most commonly reported neurological observation in early and late infantile forms (20), which is similar to that observed in the present study. Nine patients in our cohort did not exhibit neurological manifestations at the time of diagnosis. Although NPC is classically regarded as a progressive neurological disorder, the absence of neurological signs at the time of diagnosis is well recognized, particularly among patients diagnosed in the neonatal or early infantile period because of visceral manifestations such as prolonged neonatal jaundice, hepatosplenomegaly, or isolated liver disease (29). Neurological manifestations in NPC are age-dependent and may develop months to years after the onset of systemic manifestations. Therefore, the absence of neurological features at initial presentation does not exclude the diagnosis of NPC but may reflect an earlier stage of disease. A limitation of the present study is the lack of systematic longitudinal clinical follow-up. Hence, it was not possible to determine whether patients without neurological manifestations at the time of diagnosis subsequently developed any neurological signs. Nonetheless, these findings emphasize the importance of considering NPC in infants and young children presenting with unexplained hepatosplenomegaly or neonatal jaundice, even in the absence of neurological manifestations.

Interestingly, four patients in our cohort were noted to have cherry-red spots on fundus examination. Although cherry-red spots are classically associated with acid sphingomyelinase deficiency (Niemann-Pick disease types A and B), GM1 gangliosidosis, and Tay–Sachs disease (30), they have only rarely been reported in NPC (31). The occurrence of cherry-red spots in our cohort supports previous observations that retinal ganglion cell involvement may occasionally occur in NPC, thereby expanding the ophthalmological spectrum of the disease. The presence of a cherry-red spot should therefore not exclude the diagnosis of NPC, particularly when accompanied by visceral manifestations, developmental delay or neuroregression, and characteristic neurological features.

Elevated plasma chitotriosidase activity was observed in approximately half of the tested patients (52.4%), consistent with previous reports demonstrating that chitotriosidase is a nonspecific biomarker reflecting macrophage activation rather than a disease-specific marker (32, 33). In addition, reduced leukocyte acid sphingomyelinase activity was observed in ten patients while reduced β-glucosidase activity was detected in five patients despite molecular confirmation of NPC. Similar secondary reductions in lysosomal enzyme activities have been described in NPC and are thought to result from impaired lysosomal trafficking caused by intracellular cholesterol accumulation, leading to defective processing and increased degradation of mature lysosomal enzymes (34, 35). This biochemical overlap illustrates the diagnostic complexity of NPC and emphasizes that enzyme assays alone cannot reliably distinguish among lysosomal storage disorders with overlapping clinical phenotypes.

Historically, filipin staining of cultured skin fibroblasts has been regarded as the diagnostic gold standard for NPC (36). However, the requirement for invasive skin biopsy, specialized laboratory expertise, prolonged turnaround time, and limited availability has substantially restricted its routine clinical use, particularly in resource-limited settings. In the present study, filipin staining was performed in only two patients and demonstrated the characteristic intracellular accumulation of unesterified cholesterol, confirming the diagnosis. With the widespread availability of next-generation sequencing, molecular diagnosis has increasingly replaced filipin staining as the primary confirmatory test for NPC, enabling earlier and more accessible diagnosis.

The molecular findings of our cohort further illustrate the marked genetic heterogeneity of NPC. Pathogenic variants in *NPC1* accounted for 82.1% of patients, while *NPC2* variants were identified in the remaining cases, consistent with the predominance of *NPC1* reported worldwide (37). The present study identified both previously reported and novel variants in *NPC1* and *NPC2*, highlighting the marked genetic heterogeneity of NPC. This finding is consistent with reports from other populations, where a large number of private or family-specific variants have been described with relatively few recurrent mutations (22). Notably, the *NPC1* variant p.Ile1061Thr, which accounts for approximately 15%–20% of disease alleles in patients from the United States and Europe (4), was identified in only one patient in our cohort. This patient (P3) was presumed to be compound heterozygous for p.Ile1061Thr and a splice-region variant in *NPC1*. The low frequency of p.Ile1061Thr in our cohort contrasts with its high prevalence in Western populations and suggests population-specific differences in the molecular spectrum of NPC. The identification of several novel variants further expands the mutational spectrum of NPC in the Indian population and contributes to improved variant interpretation, molecular diagnosis, and genetic counselling in this underrepresented population. The interpretation of the two novel splice-region variants, c.955+3A>G and c.2373+7T>C, detected in P3 and P4 respectively warrants particular caution. The clinical phenotype and presence of another heterozygous pathogenic/likely pathogenic variant in *NPC1* (Table 2) in the same patient support an association with NPC. However, in the absence of parental segregation and RNA-based functional studies, the contribution of the two splice-region variants cannot be conclusively established. Similarly, the lack of parental segregation analysis in patients harboring two heterozygous variants is a limitation of the present study, as the phase of the variants and their occurrence in *trans* could not be established. This limits definitive assessment of their pathogenicity and biallelic involvement in NPC. Orthogonal confirmation of novel variants by Sanger sequencing was not performed in the present cohort. Although it can provide additional confirmation of NGS findings, a recent large-scale study demonstrated a high concordance between high-quality NGS variant calls and Sanger sequencing (38). Additionally in our study all variants were identified in NGS at nearly 200x depth. Nevertheless, the lack of orthogonal confirmation represents an additional limitation, particularly for novel variants. An important strength of the present study is the successful implementation of an indigenously developed smMIP-based targeted sequencing assay for molecular diagnosis. This assay enabled simultaneous detection of single nucleotide variants, small insertions/deletions, and exon-level copy number variants within a single, cost-effective workflow. The identification of two homozygous exon deletions that may have been missed by conventional sequencing approaches highlights the diagnostic advantage of incorporating copy number analysis into routine molecular testing. In countries where access to comprehensive genomic testing remains limited, such targeted approaches offer a practical and affordable strategy for improving diagnostic yield and reducing the prolonged diagnostic odyssey experienced by patients with rare genetic disorders. Future multicentre collaborative studies involving larger cohorts and standardized longitudinal follow-up are required to better define genotype-phenotype relationships, identify population-specific recurrent variants, and establish evidence-based diagnostic algorithms for NPC in the Indian population.

Two novel frameshift variants, c.2533del and c.2673dup, were predicted to generate truncated NPC1 proteins comprising 854 and 918 amino acids, respectively, compared with the 1,278-amino-acid wild-type protein. Both variants introduce premature termination codons that are predicted to result in either nonsense-mediated mRNA decay or the production of truncated proteins lacking critical functional domains (39). Structural analyses of NPC1 protein have demonstrated that the C-terminal luminal domain (CTD) is essential for maintaining the structural integrity of the protein, mediating interactions with NPC2, and facilitating intracellular cholesterol transport from the lysosomal lumen to the membrane-embedded sterol-sensing domain (40, 41). Hence, truncation of the CTD, together with the loss of the distal transmembrane helices and C-terminal lysosomal targeting region, is expected to severely impair NPC1 function, resulting in defective lysosomal cholesterol trafficking (41).

The novel missense variants identified in the present study were distributed across functionally important domains of the NPC1 protein (Figures 4, 5). The variant p.Val378Phe is located at the beginning of the middle luminal domain (MLD), a region that contributes to the structural integrity of NPC1 and mediates interdomain interactions required for intracellular cholesterol transport. The p.Ala470Pro variant is also located within the MLD, where substitution of alanine with proline is predicted to disrupt the local secondary structure because of the conformational rigidity imposed by proline. Similarly, p.Thr1093Ile is located within the C-terminal luminal domain (CTD), a highly conserved region that plays a crucial role in maintaining NPC1 structural integrity, mediating interaction with NPC2, and facilitating cholesterol transport (42). The substitution of threonine with isoleucine is predicted to alter the local conformation and stability of the CTD, potentially affecting its interaction with adjacent domains or NPC2. The p.Ala764Glu variant is located within the sterol-sensing domain (SSD), a highly conserved transmembrane region involved in cholesterol recognition and trafficking. Replacement of a small hydrophobic alanine residue with a negatively charged glutamate is predicted to alter the structural organization of the SSD and impair sterol sensing or transmembrane helix packing (43). Pathogenic variants affecting the SSD have frequently been associated with more severe clinical phenotypes in patients with NPC (44). This is likely because this domain is important for intracellular cholesterol trafficking (43).

## Conclusion

In conclusion, this study presents the largest molecularly characterized Indian cohort of Niemann-Pick disease type C and significantly expands the clinical and molecular understanding of this rare disorder in South Asia. We identified eleven novel pathogenic variants, demonstrated marked genetic heterogeneity, and highlighted the broad clinical spectrum ranging from isolated visceral disease to progressive neurological involvement. Our findings emphasize that NPC should be considered in children with unexplained hepatosplenomegaly, neonatal cholestasis, or neuroregression, even in the absence of characteristic neurological signs.

The successful implementation of an indigenous smMIP-based targeted sequencing assay enabled comprehensive and cost-effective detection of both sequence and exon-level copy number variants, supporting its utility as a practical diagnostic strategy in resource-limited settings. Early integration of molecular testing into routine clinical practice has the potential to shorten the diagnostic odyssey, improve genetic counselling and prenatal diagnosis, and facilitate timely multidisciplinary management. Collectively, these findings provide an important framework for improving the diagnosis and care of NPC in India while contributing valuable data to the global understanding of this clinically and genetically heterogeneous disorder.

## Data Availability

Raw sequence files generated from smMIP based targeted sequencing can be accessed from the European Nucleotide Archive (Project Accession: PRJEB50490) using the link https://www.ebi.ac.uk/ena/browser/search

## References

[1] Berry-Kravis E. Niemann-Pick disease, type C: diagnosis, management and disease-targeted therapies in development. Semin Pediatr Neurol. (2021) 37:100879. 10.1016/j.spen.2021.100879 33892845

[2] Vanier MT. Niemann-Pick disease type C. Orphanet J Rare Dis. (2010) 5:16. 10.1186/1750-1172-5-16 20525256 PMC2902432

[3] Dardis A Zampieri S Gellera C Carrozzo R Cattarossi S Peruzzo P. Molecular genetics of Niemann–Pick type C disease in Italy: an update on 105 patients and description of 18 NPC1 novel variants. JCM. (2020) 9(3):679. 10.3390/jcm9030679 32138288 PMC7141276

[4] Millat G Marçais C Rafi MA Yamamoto T Morris JA Pentchev PG. Niemann-Pick C1 disease: the I1061T substitution is a frequent mutant allele in patients of Western European descent and correlates with a classic juvenile phenotype. Am J Hum Genet. (1999) 65(5):1321–9. 10.1086/302626 10521297 PMC1288284

[5] Lamri A Pigeyre M Garver WS Meyre D. The extending Spectrum of NPC1-related human disorders: from Niemann–Pick C1 disease to obesity. Endocr Rev. (2018) 39(2):192–220. 10.1210/er.2017-00176 29325023 PMC5888214

[6] Saneifard H Shakiba M Alaei M Mosallanejad A Ghanefard S Yasaei M. Clinical presentation and molecular genetics of Iranian patients with Niemann-Pick type C disease and report of 6 NPC1 gene novel variants: a case series. Mol Genet Metab Rep. (2024) 40:101124. 10.1016/j.ymgmr.2024.101124 39185019 PMC11342110

[7] Sheth H Nair A Bhavsar R Kamate M Gowda VK Bavdekar A. Development, validation and application of single molecule molecular inversion probe based novel integrated genetic screening method for 29 common lysosomal storage disorders in India. Hum Genomics. (2024) 18(1):46. 10.1186/s40246-024-00613-9 38730490 PMC11088154

[8] Sheth J Nair A Bhavsar R Kamate M Gowda VK Bavdekar A. Development of national biobank for lysosomal storage disorders in India- a step towards advancing research and precision medicine. Orphanet J Rare Dis. (2026) 21(1):65. 10.1186/s13023-026-04195-8 41593770 PMC12918591

[9] Sheth J Nair A Sheth F Ajagekar M Dhondekar T Panigrahi I. Burden of rare genetic disorders in India: twenty-two years’ experience of a tertiary centre. Orphanet J Rare Dis. (2024) 19(1):295. 10.1186/s13023-024-03300-z 39138584 PMC11323464

[10] Sheth JJ Sheth FJ Oza NJ Gambhir PS Dave UP Shah RC. Plasma chitotriosidase activity in children with lysosomal storage disorders. Indian J Pediatr. (2010) 77(2):203–5. 10.1007/s12098-009-0249-0 19936666

[11] Kruth HS Vaughan M. Quantification of low density lipoprotein binding and cholesterol accumulation by single human fibroblasts using fluorescence microscopy. J Lipid Res. (1980) 21(1):123–30. 10.1016/S0022-2275(20)39846-1 6986448

[12] Sheth JJ Sheth FJ Oza N. Niemann-Pick type C disease. Indian Pediatr. (2008) 45(6):505–7. PMID: 18599941 18599941

[13] Sheth J Mistri M Sheth F Shah R Bavdekar A Godbole K. Burden of lysosomal storage disorders in India: experience of 387 affected children from a single diagnostic facility. JIMD Rep. (2014) 12:51–63. 10.1007/8904_2013_244 23852624 PMC3897787

[14] Sheth J Mistri M Bhavsar R Sheth F Kamate M Shah H. Lysosomal storage disorders in Indian children with neuroregression attending a genetic center. Indian Pediatr. (2015) 52(12):1029–33. 10.1007/s13312-015-0768-x 26713986

[15] Li H Durbin R. Fast and accurate short read alignment with burrows–wheeler transform. Bioinformatics. (2009) 25(14):1754–60. 10.1093/bioinformatics/btp324 19451168 PMC2705234

[16] McKenna A Hanna M Banks E Sivachenko A Cibulskis K Kernytsky A. The genome analysis toolkit: a MapReduce framework for analyzing next-generation DNA sequencing data. Genome Res. (2010) 20(9):1297–303. 10.1101/gr.107524.110 20644199 PMC2928508

[17] Smedley D Jacobsen JOB Jäger M Köhler S Holtgrewe M Schubach M. Next-generation diagnostics and disease-gene discovery with the exomiser. Nat Protoc. (2015) 10(12):2004–15. 10.1038/nprot.2015.124 26562621 PMC5467691

[18] Rodrigues CHM Pires DEV Ascher DB. Dynamut2 : assessing changes in stability and flexibility upon single and multiple point missense mutations. Protein Sci. (2021) 30(1):60–9. 10.1002/pro.3942 32881105 PMC7737773

[19] Jubb HC Higueruelo AP Ochoa-Montaño B Pitt WR Ascher DB Blundell TL. Arpeggio: a web server for calculating and visualising interatomic interactions in protein structures. J Mol Biol. (2017) 429(3):365–71. 10.1016/j.jmb.2016.12.004 27964945 PMC5282402

[20] Bolton SC Soran V Marfa MP Imrie J Gissen P Jahnova H. Clinical disease characteristics of patients with Niemann-Pick disease type C: findings from the international Niemann-Pick disease registry (INPDR). Orphanet J Rare Dis. (2022) 17:51. 10.1186/s13023-022-02200-4 35164809 PMC8842861

[21] Tängemo C Weber D Theiss S Mengel E Runz H. Niemann-Pick type C disease: characterizing lipid levels in patients with variant lysosomal cholesterol storage. J Lipid Res. (2011) 52(4):813–25. 10.1194/jlr.P013524 PMC3284170 21245028

[22] Touma L Labrecque M Tetreault M Duquette A. Identification and classification of rare variants in NPC1 and NPC2 in Quebec. Sci Rep. (2021) 11(1):10344. 10.1038/s41598-021-89630-5 33990640 PMC8121778

[23] Guatibonza Moreno P Pardo LM Pereira C Schroeder S Vagiri D Almeida LS. At a glance: the largest Niemann-Pick type C1 cohort with 602 patients diagnosed over 15 years. Eur J Hum Genet. (2023) 31(10):1108–16. 10.1038/s41431-023-01408-7 37433892 PMC10545733

[24] Sheth J Joseph JJ Shah K Muranjan M Mistri M Sheth F. Pulmonary manifestations in Niemann-Pick type C disease with mutations in NPC2 gene: case report and review of literature. BMC Med Genet. (2017) 18(1):5. 10.1186/s12881-017-0367-x 28095804 PMC5240394

[25] Patterson MC Mengel E Vanier MT Moneuse P Rosenberg D Pineda M. Treatment outcomes following continuous miglustat therapy in patients with Niemann-Pick disease type C: a final report of the NPC registry. Orphanet J Rare Dis. (2020) 15(1):104. 10.1186/s13023-020-01363-2 32334605 PMC7183679

[26] Evans WRH Hendriksz CJ. Niemann-Pick type C disease—the tip of the iceberg? A review of neuropsychiatric presentation, diagnosis and treatment. BJPsych Bull. (2017) 41(2):109–14. 10.1192/pb.bp.116.054072 28400970 PMC5376728

[27] Mavridou I Dimitriou E Vanier MT Vilageliu L Grinberg D Latour P. The Spectrum of Niemann-Pick type C disease in Greece. JIMD Rep. (2017) 36:41–8. 10.1007/8904_2016_41 28105569 PMC5680277

[28] Geberhiwot T Moro A Dardis A Ramaswami U Sirrs S Marfa MP. Consensus clinical management guidelines for niemann-pick disease type C. Orphanet J Rare Dis. (2018) 13:50. 10.1186/s13023-018-0785-7 29625568 PMC5889539

[29] Imrie J Dasgupta S Besley GTN Harris C Heptinstall L Knight S. The natural history of Niemann-Pick disease type C in the UK. J Inherit Metab Dis. (2007) 30(1):51–9. 10.1007/s10545-006-0384-7 17160617

[30] Kivlin JD Sanborn GE Myers GG. The cherry-red spot in Tay-Sachs and other storage diseases. Ann Neurol. (1985) 17(4):356–60. 10.1002/ana.410170409 4004157

[31] Chen H Chan AY Stone DU Mandal NA. Beyond the cherry-red spot: ocular manifestations of sphingolipid-mediated neurodegenerative and inflammatory disorders. Surv Ophthalmol. (2014) 59(1):64–76. 10.1016/j.survophthal.2013.02.005 24011710 PMC3864975

[32] Michelakakis H Dimitriou E Labadaridis I. The expanding spectrum of disorders with elevated plasma chitotriosidase activity: an update. J Inherit Metab Dis. (2004) 27(5):705–6. 10.1023/b:boli.0000043025.17721.fc 15669690

[33] Ries M Schaefer E Lührs T Mani L Kuhn J Vanier MT. Critical assessment of chitotriosidase analysis in the rational laboratory diagnosis of children with Gaucher disease and Niemann-Pick disease type A/B and C. J Inherit Metab Dis. (2006) 29(5):647–52. 10.1007/s10545-006-0363-3 16972172

[34] Devlin C Pipalia NH Liao X Schuchman EH Maxfield FR Tabas I. Improvement in lipid and protein trafficking in Niemann-Pick C1 cells by correction of a secondary enzyme defect. Traffic. (2010) 11(5):601–15. 10.1111/j.1600-0854.2010.01046.x 20412078 PMC3000737

[35] Lo SM McNamara J Seashore MR Mistry PK. Misdiagnosis of Niemann-Pick disease type C as Gaucher disease. J Inherit Metab Dis. (2010) 33(0 3):429–33. 10.1007/s10545-010-9214-3 20882348 PMC3053412

[36] Vanier MT Latour P. Laboratory diagnosis of Niemann-Pick disease type C: the filipin staining test. Methods Cell Biol. (2015) 126:357–75. 10.1016/bs.mcb.2014.10.028 25665455

[37] McKay Bounford K Gissen P. Genetic and laboratory diagnostic approach in Niemann Pick disease type C. J Neurol. (2014) 261(Suppl 2):569–75. 10.1007/s00415-014-7386-8 PMC4141153 25145893

[38] Beck TF Mullikin JC; NISC Comparative Sequencing Program, Biesecker LG. Systematic evaluation of sanger validation of next-generation sequencing variants. Clin Chem. (2016) 62(4):647–54. 10.1373/clinchem.2015.249623 26847218 PMC4878677

[39] Kurosaki T Maquat LE. Nonsense-mediated mRNA decay in humans at a glance. J Cell Sci. (2016) 129(3):461–7. 10.1242/jcs.181008 26787741 PMC4760306

[40] Gong X Qian H Zhou X Wu J Wan T Cao P. Structural insights into the Niemann-Pick C1 (NPC1)-mediated cholesterol transfer and ebola infection. Cell. (2016) 165(6):1467–78. 10.1016/j.cell.2016.05.022 27238017 PMC7111323

[41] Li X Wang J Coutavas E Shi H Hao Q Blobel G. Structure of human Niemann–Pick C1 protein. Proc Natl Acad Sci USA. (2016) 113(29):8212–7. 10.1073/pnas.1607795113 27307437 PMC4961162

[42] Li X Lu F Trinh MN Schmiege P Seemann J Wang J. 3.3 Å structure of Niemann–Pick C1 protein reveals insights into the function of the C-terminal luminal domain in cholesterol transport. Proc Natl Acad Sci U S A. (2017) 114(34):9116–21. 10.1073/pnas.1711716114 28784760 PMC5576846

[43] Dubey V Bozorg B Wüstner D Khandelia H. Cholesterol binding to the sterol-sensing region of Niemann Pick C1 protein confines dynamics of its N-terminal domain. PLoS Comput Biol. (2020) 16(10):e1007554. 10.1371/journal.pcbi.1007554 33021976 PMC7537887

[44] Las Heras M Szenfeld B Ballout RA Buratti E Zanlungo S Dardis A. Understanding the phenotypic variability in Niemann-Pick disease type C (NPC): a need for precision medicine. npj Genom Med. (2023) 8(1):21. 10.1038/s41525-023-00365-w 37567876 PMC10421955

[45] Biesecker LG Harrison SM, ClinGen Sequence Variant Interpretation Working Group. The ACMG/AMP reputable source criteria for the interpretation of sequence variants. Genet Med. (2018) 20(12):1687–8. 10.1038/gim.2018.42 29543229 PMC6709533

[46] Richards S Aziz N Bale S Bick D Das S Gastier-Foster J. Standards and guidelines for the interpretation of sequence variants: a joint consensus recommendation of the American College of Medical Genetics and Genomics and the Association for Molecular Pathology. Genet Med. (2015) 17(5):405–24. 10.1038/gim.2015.30 25741868 PMC4544753

