# Supplementary material for: Clinical and molecular spectrum of Niemann-Pick disease type C patients in India
Source: Front Pediatr. 2026 Sep 4;14:1950899. doi: 10.3389/fped.2026.1950899 (PMC13586024; doi:10.3389/fped.2026.1950899)
Supplement: Supplementary file 1 [file Datasheet1.docx]

**Methodology**

**Sanger sequencing**

Genomic DNA extracted from one patient (P24) was subjected to PCR for amplification of exon-1 of the *NPC2* gene using primers (FP: 5’-ACAGGTTTGTCTTGTGACCG-3’; RP: 5’-AGCCCCAGGGGTCTCAG-3’). The cycling conditions were as follows: initial denaturation (94°C; 5 min), followed by 35 cycles of denaturation (94°C; 30 s), annealing (61°C; 30 s), elongation (72°C; 1 min), and final elongation (72°C; 5 min). Following this, the PCR products were purified using Exo-SAP-IT™ (USB Corporation, USA) and subjected to di-deoxy chain termination protocol using BigDye Terminator v3.1 cycle sequencing kit (Thermo Fisher Scientific, USA). Capillary electrophoresis was performed using an automated sequencer SeqStudio (Applied Biosystem, USA). Sequences were assessed by comparing with the hg19/GRCh37 genomic reference sequence of *NPC2* gene using NCBI-BLAST (<https://blast.ncbi.nlm.nih.gov/Blast.cgi>).

**Prediction of structural effects of the variants**

The protein sequence of NPC1 in FASTA format was retrieved from the UniProt database (UniProt ID: O15118). The three-dimensional (3D) structure, with a resolution of 3.0 Å, was obtained from the Protein Data Bank (PDB) (PDB ID: 6W5S). This structure was selected because it is currently the highest resolution full-length model of NPC1 available. All mutation sites analyzed were located within resolved regions of the structure. Additionally, 6W5S has been widely used as a reference structure in computational studies of NPC1, establishing it as a validated model. The amino acid numbering of the sequence and structure was correlated using the Pairwise Sequence Alignment tool (1). The Mutagenesis Wizard function of PyMOL was used to introduce specific amino acid changes.

The effects of amino acid substitutions on protein stability and dynamics were assessed using DynaMut2 (2), while pathogenicity was further evaluated using AlphaMissense and MutPred2 (3,4). Evolutionary conservation of affected residues was examined using multiple sequence alignment generated through AlphaFold2 implemented in ColabFold (5,6). Interatomic interactions surrounding variant residues were analyzed using the Arpeggio server (7). Three-dimensional structures of wildtype and mutant NPC1 proteins were visualized using PyMOL.

References

1.	Bittrich S, Segura J, Duarte JM, Burley SK, Rose Y. RCSB protein Data Bank: exploring protein 3D similarities via comprehensive structural alignments. Elofsson A, editor. Bioinformatics. 2024 Jun 3;40(6):btae370. doi:10.1093/bioinformatics/btae370

2.	Rodrigues CHM, Pires DEV, Ascher DB. DynaMut2 : Assessing changes in stability and flexibility upon single and multiple point missense mutations. Protein Science. 2021 Jan;30(1):60–9. doi:10.1002/pro.3942

3.	Pejaver V, Urresti J, Lugo-Martinez J, Pagel KA, Lin GN, Nam HJ, et al. Inferring the molecular and phenotypic impact of amino acid variants with MutPred2. Nat Commun. 2020 Nov 20;11(1):5918. doi:10.1038/s41467-020-19669-x

4.	Tordai H, Torres O, Csepi M, Padányi R, Lukács GL, Hegedűs T. Analysis of AlphaMissense data in different protein groups and structural context. Sci Data. 2024 May 14;11(1):495. doi:10.1038/s41597-024-03327-8

5.	Jumper J, Evans R, Pritzel A, Green T, Figurnov M, Ronneberger O, et al. Highly accurate protein structure prediction with AlphaFold. Nature. 2021 Aug 26;596(7873):583–9. doi:10.1038/s41586-021-03819-2

6.	Mirdita M, Schütze K, Moriwaki Y, Heo L, Ovchinnikov S, Steinegger M. ColabFold: making protein folding accessible to all. Nat Methods. 2022 Jun;19(6):679–82. doi:10.1038/s41592-022-01488-1

7.	Jubb HC, Higueruelo AP, Ochoa-Montaño B, Pitt WR, Ascher DB, Blundell TL. Arpeggio: A Web Server for Calculating and Visualising Interatomic Interactions in Protein Structures. Journal of Molecular Biology. 2017 Feb;429(3):365–71. doi:10.1016/j.jmb.2016.12.004
